# PsychOpen CAMA: Publication of community‐augmented meta‐analyses in psychology

**DOI:** 10.1002/jrsm.1536

**Published:** 2021-11-17

**Authors:** Tanja Burgard, Michael Bosnjak, Robert Studtrucker

**Affiliations:** ^1^ ZPID – Leibniz Institute for Psychology Trier Germany; ^2^ Department of Psychological Research Methods, University of Trier Trier Germany

**Keywords:** CAMA, cumulative research, meta‐analysis, open synthesis, replicability, re‐usability

## Abstract

To enable optimal decision‐making based on the best evidence available, open syntheses are called for. To make data accessible and comprehensible even for decision‐makers without proficient knowledge in meta‐analysis, a graphical user interface (GUI) provides flexible data visualizations including interpretation aids. Moreover, due to a growing number of research findings, efficient and easy updating of meta‐analyses is crucial to prevent waste in research. One label for a concept to meet these needs is community‐augmented meta‐analysis (CAMA). The research community at the one hand feeds the data repository of a CAMA with new data and on the other hand benefits from easy access to data and meta‐analyses on a GUI. PsychOpen CAMA has been released recently to serve the psychological research community as a whole by covering a broad scope of potential research domains. PsychOpen CAMA relies on a web application with an OpenCPU server for the R calculations. To achieve interoperability of different datasets with the analysis functions used in PsychOpen CAMA, a template for meta‐analytic data and machine‐readable metadata are used. In the future, the automation of workflows, flexibility of analysis options, and the scope of the platform will be further developed by making use of synergies with other resources and tools at ZPID. The article provides an overview on the rationale for the necessity of open syntheses and the CAMA approach, as well as a presentation of the architecture, user interface, functionalities and future challenges of PsychOpen CAMA.

**Highlights**

**What is already known**

Due to a growing number of research findings, accessibility of data and technical solutions to enable the timely and flawless reproduction and updating of meta‐analyses are needed to provide the best available evidence for theory building/refinement and practical decision‐making. An approach for dynamic and reusable meta‐analyses is community‐augmented meta‐analysis (CAMA).

**What is new**

PsychOpen CAMA is the first general purpose web application to serve the psychological research community by providing a repository with standardized meta‐analytic data and a graphical user interface to use data from this repository for meta‐analytic calculations and visualizations.

**Potential impact**

By using PsychOpen CAMA, meta‐analytic data can be reused by the research community and data curators to verify, modify, and update meta‐analyses in psychology and neighboring fields.

## 1 INTRODUCTION

Empirical evidence in psychology serves do develop and to refine theories and is crucial for decision‐making in many applied areas, such as the effectiveness of psychotherapies,^1^ the effects of interventions on health behavior,^2^ or the influence of workplace conditions on the mental health of employees.^3^ To facilitate theory building, theory refinement, and the translation of empirical findings into practice, research syntheses have to be timely and accessible for different audiences. Due to a large and growing number of published findings,^4^ the results of meta‐analyses may outdate rapidly.^5^ Updating existing meta‐analyses is often time‐consuming, as relevant information on the methodology of the meta‐analysis or the complete meta‐analytic data with the information from primary studies already collected cannot be accessed for an efficient re‐use.^6^

In order to optimize evidence‐based theory‐development and decision‐making, and to enable the timely and efficient updating of evidence, the principles of open science^7^ and FAIR (Findable, Accessible, Interoperable, Re‐usable) data^8^ can serve as guidelines for future evidence syntheses and are summarized in the first section. A concept that meets the requirements of open and FAIR synthesis is presented in the second section: Community‐Augmented Meta‐Analysis (CAMA). In the third section, a freely available CAMA system serving the psychological research community and related disciplines (e.g., the life and management sciences) is introduced: PsychOpen CAMA. Finally, future challenges and planned developments of the platform are discussed.

## 2 REQUIREMENTS OF OPEN SYNTHESIS AND FAIR DATA

To enable reproducibility of meta‐analyses, open meta‐analytic data^9^ are required. However, open data is not sufficient. According to the principles of the Open Science Movement,^7^ open syntheses should additionally provide information on the methodology in sufficient detail to allow verification and replicability, open programming code and tools, and open access to all relevant information.^10^ This would allow the research community to replicate, re‐use, and update meta‐analyses more efficiently and prevent ambiguities and questionable practices in research, as literature selection and data collection could rely on sufficient information on previous work. Research infrastructures are needed to facilitate the accumulation of evidence by fostering FAIR data sharing. FAIR data support the readability of data both for machines and for humans.^8^

Especially for evidence syntheses, the findability and accessibility for humans is crucial to make summarized evidence available to optimize decision‐making. To serve the purpose of providing information in practical contexts, the comprehensibility of results is of high relevance. A graphical user interface (GUI) providing visualizations including interpretation aids can enable users without proficient knowledge of meta‐analytic methods to use meta‐analytic data to get an overview on the evidence on a research question.^11^ Plain Language Summaries giving a summary of the existing evidence, in the tradition of Cochrane reviews,^12^ can complement the GUI to make scientific knowledge accessible for decision‐makers and the public.

To create interoperability of data, metadata, and tools, common data standards have to be defined. The aim of interoperability is to enable machines and technical tools to understand and analyze new data automatically. Especially for evidence syntheses, interoperability is of high relevance, as it facilitates and accelerates efficient evidence accumulation for a timely integration of new research findings in synthesized evidence.

For researchers with further interest in a published meta‐analysis, data access, information about the providence and licensing of the data, as well as a thorough documentation of the underlying methodology is crucial to be able to replicate or re‐use the data. Available analysis scripts and technical tools facilitate the replication of published results. Above this, existing data resources can be used for novel purposes, as subgroup analyses, or the modification of methodological decisions, as estimation method and modeling choices.^9^

Data infrastructures adhering to the FAIR data principles thus have the potential to improve the efficiency of collaborative evidence collection and at the same time, increase the usability and accessibility of information for decision‐makers and the public. In the following, we present the architecture, user interface, and functionalities of a platform for CAMA. A CAMA platform can serve as a technical infrastructure to facilitate open synthesis and as a common tool for the research community.

## 3 COMMUNITY‐AUGMENTED META‐ANALYSIS

Actually, a concept for a platform that meets the requirements of open synthesis and FAIR data already exists. There are slightly differing forms that have been suggested including living,^13^ dynamic,^14^ or cloud‐based meta‐analysis.^11^ Braver et al^15^ describe an approach called continuously cumulating meta‐analysis (CCMA) to incorporate and evaluate new replication attempts to existing meta‐analyses. All of these approaches have in common that they aim at accumulating scientific results to keep evidence up‐to‐date.

We use the term community‐augmented meta‐analysis, CAMA for short,^16^ to describe a platform providing an open repository for meta‐analytic data and a GUI for meta‐analytic analysis tools. A CAMA is supposed to facilitate and foster the accumulation of evidence by providing a user‐friendly infrastructure to the research community. Existing systems in psychology have been reviewed and presented in a previous article.^17^ In essence, four systems were compared: The crowdsourced platform Curate Science^18^ allows the permanent curation of findings by the research community and enables a systematic evaluation of empirical research, primarily in cognitive and social psychology. The project Cochrane living systematic reviews (LSRs)^19^ suggests continuous updating of reviews with a high priority for health decision making or a high likelihood of new evidence to affect the conclusions of the review. LSRs are continuously updated and published in the database of systematic reviews.

MetaBUS^20^ and metalab^21^ both offer shiny webapps as responsive interfaces to reproduce meta‐analyses. MetaBUS relies on semi‐automatic data collection for continuous curation of research findings in management and applied psychology. In the case of metalab, the research community curates the meta‐analyses in the field of language acquisition and cognitive development. The three main distinctions between PsychOpen CAMA and the previous systems are the general scope addressing all domains of psychology, the architecture as a PHP web application for better system stability and scalability, and the long‐term integration with other services offered by ZPID—Leibniz Institute for Psychology for a more sustainable and efficient data curation and additional user benefit.

**FIGURE 1:**
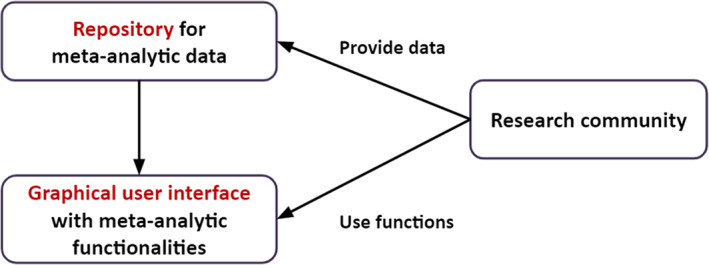
The basic concept of community‐augmented meta‐analysis systems [Colour figure can be viewed at wileyonlinelibrary.com]

Figure 1 illustrates the essence of a CAMA system. On the one hand, it provides a repository for meta‐analytic data. The research community can provide complete meta‐analyses or update existing ones in this repository. On the other hand, a GUI with meta‐analytic functionalities is provided. The GUI is connected to the repository. Thus, users are enabled to easily access the data in the repository and request meta‐analytic outputs by using the functions in the point‐and‐click interface.

The role of the infrastructure provider is to set standards for the data submitted to the repository and to store the data according to these standards. The functionalities on the GUI are interoperable with these data and thus, as meta‐analytic datasets are augmented or new meta‐analyses are implemented, analysis outputs can be requested by users and are automatically available on the GUI. The platform thus serves as a dynamic resource enabling the research community to keep the state of research updated and accumulate knowledge continuously by providing a common language for the data.

To sum up, a CAMA adheres to the requirements of FAIR data by making research results findable, complete datasets accessible, ensuring interoperability of data and analysis scripts, and thus, making data reusable.^22^

## 4 FROM META‐ANALYTIC DATA TO DYNAMIC SYNTHESIS IN PSYCHOPEN CAMA

The Leibniz Institute for Psychology (ZPID) provides PsychOpen CAMA as a freely available platform for meta‐analyses here https://cama.psychopen.eu/. This service aims to serve the psychological research community by covering a broad scope of potential research domains. Meta‐analyses can be published via the platform to become accessible to and expandable by the community.

**FIGURE 2:**
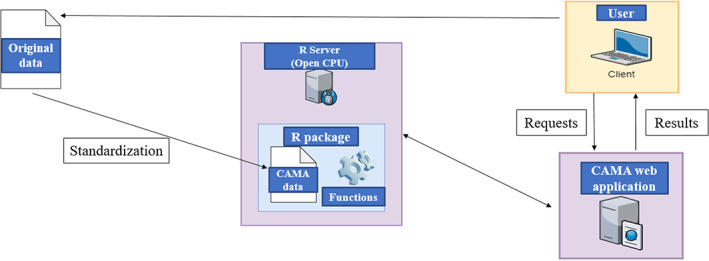
Architecture of PsychOpen community‐augmented meta‐analysis [Colour figure can be viewed at wileyonlinelibrary.com]

As Figure 2 shows, original meta‐analytic data from users is standardized to adhere to the structure and naming of CAMA data. Standardized data becomes part of the self‐maintained R package that also contains all functions needed for analysis requests offered on the GUI. For the meta‐analytic computations and visualizations in the self‐maintained package, functions from the following R packages were used: metafor,^23^ dmetar,^24^ metaviz,^25^ meta.^26^

PsychOpen CAMA relies on a web application. The R calculations that are requested from the user by choosing a dataset and a desired meta‐analytic output in the application are forwarded to an OpenCPU server (https://www.opencpu.org/). There, the analyses are executed using the dataset and function of interest from the R package. The resulting outputs are embedded in the web application and thus displayed to the user. The use of the R server improves the system stability and scalability of the tool according to the number of users compared to commonly used R shiny architectures. Furthermore, the web application programmed in PHP is more flexible in the design and crucial for the technical connection to other ZPID services.

### 4.1 Interoperability: Data standardization and metadata

**FIGURE 3:**
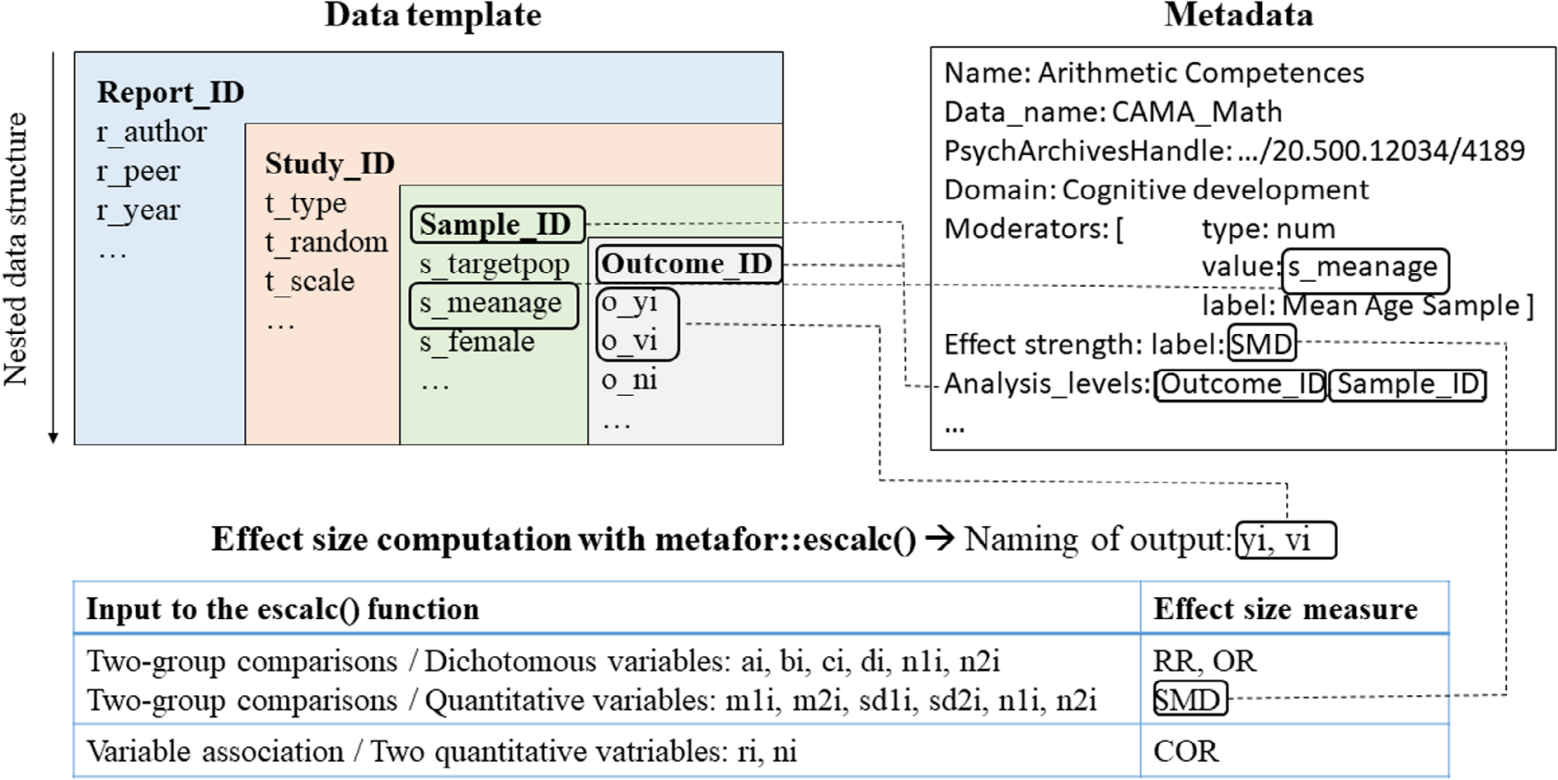
Interconnections of data and metadata in PsychOpen community‐augmented meta‐analysis [Colour figure can be viewed at wileyonlinelibrary.com]

Interoperability enables operational processes and information exchange between different systems. Optimally, standardized identifiers and metadata for all data and digital objects allow for an automated access and use of data by humans and machines.^27^ Figure 3 provides an overview on the interconnections between meta‐analytic datasets, machine‐readable metadata, and meta‐analytic functions to achieve interoperability of different datasets used in PsychOpen CAMA.

The data template for CAMA datasets can be found under the menu item “Data Contribution” in the web application. Additional information on how to contribute to PsychOpen CAMA, as well as information on filling in data in the template is also provided there. As a first orientation for a template for basic meta‐analytic information on report, study, and sample, the spreadsheet of metalab^16^ served as a starting point. As the meta‐analyses in metalab are all located in the domain of language acquisition and cognitive development, adaptations for other fields of research are necessary. As it is not possible to include each moderator variable that might be relevant in any field, the template is kept rather generic and leaves space for specific adaptations in the form of adding relevant moderators that are not included in the basic template.

As the schematic illustration of the data template in Figure 3 illustrates, the template of PsychOpen CAMA suggests the collection of data on different levels. There may be dependencies in the outcome measures of meta‐analyses. This might occur, if the effect of an intervention is measured using multiple outcomes, for example competences in different domains. If multiple outcomes are measured using the same study sample, results within a sample might be more similar than between samples. Not accounting for this potential covariance of the outcome measures from the same sample can bias statistical inferences.^28^ Data do not have to be nested necessarily. In some meta‐analyses, there might only be one outcome measure per sample and report. However, the structure and variable naming enable to distinguish the information levels of the variables and—in case of dependencies—automatically trigger the use of a multilevel model in the analysis scripts.

The variable names of the outcome data follow the naming of potential measures serving as inputs to compute effect sizes with the function metafor::escalc.^23^ In Figure 3, the use of arguments for this function for some of the most frequently used effect size measures is depicted. As PsychOpen CAMA operates mainly with metafor and effect sizes are computed using metafor::escalc, the various outcome information potentially given in a report coded for a meta‐analysis is named according to the inputs to metafor::escalc. The resulting effect size of interest and the corresponding variance are automatically labeled “yi” and “vi”. This terminology is therefore also used in PsychOpen CAMA.

To ensure that recoding did not affect the results, analyses of the original publication or analyses with the original dataset are replicated with the restructured CAMA dataset. The outputs from both analyses should be the same. If there are inconsistencies, these are resolved between the CAMA team and the data provider.

The naming of metafor is also used in the metadata as depicted in Figure 3. For example, the kind of effect size measure has to be given for each meta‐analysis. The options for the “measure” argument in metafor::escalc are used in various functions in metafor and are crucial to ensure the use of the correct estimation formulas. Therefore, following the standards of metafor in this case is also reasonable. The metadata of a dataset also contains information on relevant moderator variables. This is necessary to provide the desired labeling of a moderator in the web application and to automatically consider whether a moderator is numeric or categorical in a meta‐regression. To allow for an automated use of a multilevel model, the nesting of the data is provided in the metadata. If analysis levels are defined there, these are used as random parameters in a multilevel model.

Finally, the metadata also contains bibliographic and methodological information, as the DOIs of the primary studies of a meta‐analysis, the research question, or the inclusion criteria used in a meta‐analysis. The metadata thus also serves the purpose of documentation of the datasets.

### 4.2 An empirical example of a CAMA

In the first version released, PsychOpen CAMA provides a GUI, offering the user easy access to the results of 15 meta‐analytic datasets (November 2021) from five different data providers. Next to student assistants, these data providers also acted as test users of the system to ensure an adequate representation of their meta‐analyses on the platform.

In the following, we look at one of these datasets in more detail, namely “Correlation of Narcissism and Machiavellianism”,^29^ a meta‐analysis in the field of the Dark Triad of personality examining the interrelation between two of the components of the Dark Triad. The data submission process, as well as the functionalities of the intuitive and responsive point‐and‐click tool to explore the data will be presented in the following empirical example.

The data provider acted as a test user of the CAMA system and in close collaboration with the PsychOpen CAMA team. Data and R scripts were provided the CAMA team recoded the data and replicated the basic analyses in the scripts with the original and the restructured data. Inconsistencies and minor differences in the results were discussed and resolved. All information needed to fill in the metadata for a thorough methodological documentation were handed over by the data provider. The CAMA team filled in this information appropriately in a json‐file and implemented the CAMA on the dataset on a test server that the data provider had access to. Thus, data and results were again checked within the platform by the original provider. Finally, the CAMA dataset and a corresponding codebook were stored in our archive for digital research objects in psychology, PsychArchives,^30^ and became available in the live version of PsychOpen CAMA. Future data providers are expected to prepare their datasets according to the template themselves. However, guidance and support will still be provided by the CAMA team.

For the user of the platform, the menu item “Data” contains a thorough documentation, including bibliographic and methodological information, as well as links to primary studies included in the meta‐analyses, and a complete data table. In the case of the meta‐analysis on the interrelation of narcissism and machiavellianism, the user for example finds out that no multilevel meta‐analysis is used, as only one effect size per sample was used to ensure statistical independence of the outcomes. Overall, the meta‐analysis contains 172 effect sizes (November 2021).

A data exploration tool provides a quick overview on the univariate distributions of the correlation between narcissism and machiavellianism and the bivariate and trivariate distributions with potentially relevant moderator variables. For example, the user can select the scale type as a moderator. The resulting violinplots depict the distribution of the correlations for individual and composite scales. The plot suggests that in case of composite scales, higher correlations between the two components of the Dark Triad result. If the user is interested in two numeric moderators, for example mean age of the sample and publication year, a scatterplot matrix displays all pairwise interrelations of the correlation of interest, the mean age, and the publication year.

Basic meta‐analytic outputs can be found under the item “Analyses.” A random effects meta‐analysis is presented. If the effect size is a correlation as in this case, the meta‐analysis is conducted with z‐transformed values and the results are back‐transformed to Pearson's *r* for interpretation.^31^ The mean correlation resulting in the meta‐analysis on narcissism and machiavellianism is thus 0.48, speaking for a medium to strong interrelation of the two components. As a graphical display of the meta‐analytic results, a forest plot and a cumulative forest plot are provided.

The responsive GUI also allows to conduct meta‐regressions with each up to two moderator variables. As the exploratory analyses suggested that higher correlations are measured with composite scales, this could be examined in a meta‐regression by choosing scale type as a moderator. The results of the meta‐regression indeed speak for a significantly higher interrelation if a composite scale is used. The user however should always keep in mind that this does not necessarily mean that there is a causal relation between the scale type and the interrelation. The tool enables to examine datasets interactively. However, the output does not provide sufficient information on the role of potential further influencing factors or the statistical power of the meta‐regression. Additional educational material is needed to create awareness for the potential limits of the tool. The topic of meta‐analytic education in PsychOpen CAMA will be further discussed below.

**FIGURE 4:**
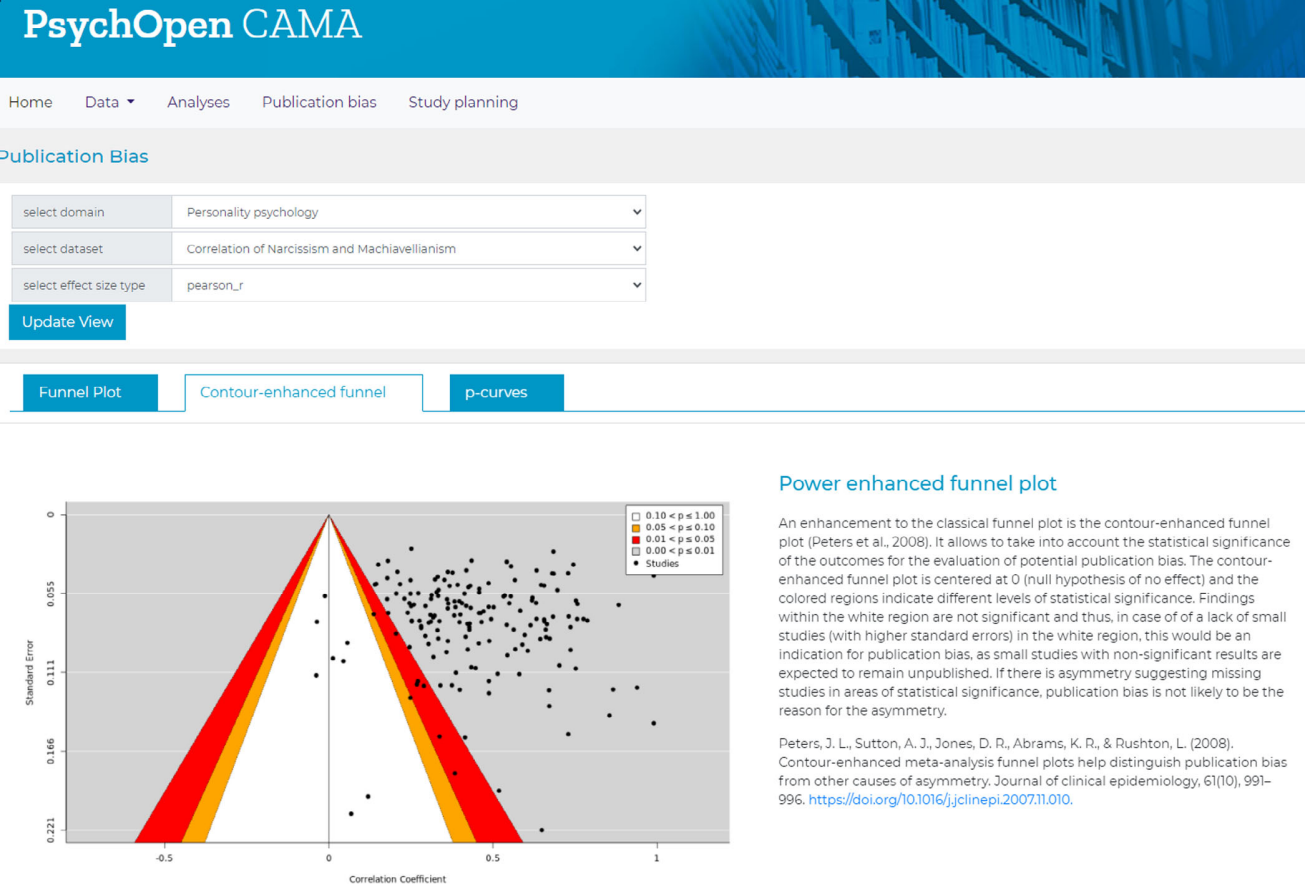
Screenshot of a contour‐enhanced funnel plot within the menu item publication bias [Colour figure can be viewed at wileyonlinelibrary.com]

In Figure 4, one of the outputs to assess potential publication bias is displayed, the contour‐enhanced funnel plot. A classical funnel plot, the results of an Egger's test,^32^ as well as p‐curve analysis^33^ are also available in this context to give the user the opportunity to assess the evidential value and potential bias of a meta‐analysis using different tools. For the meta‐analysis on narcissism and machiavellianism, no indication of publication bias or p‐hacking can be found and almost all of the 172 individual effect sizes found a significant correlation between the two components.

Finally, a study planning tool allows one to conduct a prospective power analysis for a potential new study on the research questions of the selected meta‐analysis. Therefore, the meta‐analytic estimate is assumed as the true underlying effect size. The sample size and desired significance level are chosen by the user. The tool calculates the expected power of the prospective study, as well as a necessary sample size to achieve a power of 80%. This provides a quick indication of how large a study in a certain domain needs to be to achieve sufficient statistical power and may thus guide researchers in planning new studies. For our example CAMA the tool reveals that a study with 50 participants would find a significant effect with a probability of almost 60% with a regular significance level of 5%. To achieve 80% power, a sample size of *n* = 81 would be needed.

Despite the relatively strong correlation and the robustness of the findings, a small study thus would probably not even find an overall effect. This underlines the need to accumulate evidence leading back to data contribution. In PsychOpen CAMA, existing meta‐analyses can be updated by the community. To add evidence from new studies to a meta‐analysis, the dataset of interest can be downloaded from PsychArchives. With the help of the corresponding codebook and the information to fill in the CAMA template, the additional information can be added to the dataset. The extended data can then be submitted for an update. Concerning the relevance and quality of the additional data, next to the CAMA team the original data provider as an expert for the meta‐analysis can also be involved in the evaluation, if he or she is willing to.

### 4.3 Education on meta‐analyses in PsychOpen CAMA

PsychOpen CAMA is also suited to serve educational purposes in the field of meta‐analysis. Interpretation aids to each output make the results comprehensible, even for scientific laypersons. Within the menu item “Data,” the user gets an overview on methodological decisions in research syntheses and learns how a meta‐analytic dataset may look like. The use of the data template is explained in more detail by providing examples and a collection of Frequently Asked Questions.

The random effects meta‐analyses and graphical outputs in the item “Analyses” are produced with the metafor package in R. A detailed description of the statistical coefficients is given next to the output to give the user the opportunity to understand the statistics behind and to draw conclusions from the results. For the outputs on publication bias, the rationale of the graphical devices is explained and further literature on the methods presented is also provided. Additional educational material on the interpretation of moderating effects and on potential limits of the tool is planned. For instance, users should be informed that there might be potential further influencing factors not included in the meta‐analysis and that moderator analyses might also suffer from low statistical power.^34^

In order to increase the accessibility of analyses in PsychOpen CAMA for patients and the public, plain language summaries will be made available for each published meta‐analysis. For this purpose, expertise from the PLan Psy project^35^ at ZPID will be used. The project team is developing evidence‐based guidelines for the preparation of plain language summaries of psychological meta‐analyses. Therefore, experiments will investigate how characteristics of plain language summaries, as approaches to explain statistical outcomes, affect the comprehensibility of the summaries. The PLan Psy project already started to work with PsychOpen CAMA and, next to the data providers, acted as pilot testers of the systems. Educational material on meta‐analyses and PsychOpen CAMA in collaboration with PLan Psy are already under way.

For advanced users and experts within the research community, especially the further development of PsychOpen CAMA to enable more advanced meta‐analytic methods is of interest. However, this has to be accompanied by educational material and training opportunities. Therefore, detailed descriptions of the outputs and their meanings within the user interface, additional tutorial videos and workshops targeted at the psychological research community (e.g., at relevant conferences or training institutes) should be offered.

## 5 FUTURE CHALLENGES FOR PSYCHOPEN CAMA

As a central research infrastructure institute for psychology, ZPID has resources and tools to provide users assistance with data submission and updating of data. Furthermore, the benefit of PsychOpen CAMA can be increased by giving users far‐reaching and flexible analysis options. By using already existing resources within ZPID, we can reduce manual effort and simultaneously increase potential applications for the user. In the following, we will describe the planned synergies of PsychOpen CAMA with other ZPID services, concerning data acquisition, data analysis, and the methodological and topical scope of the service.

### 5.1 Data acquisition and updating: Crowdsourcing and automation

The continuous maintenance of a CAMA repository is both time‐ and labor‐intensive. The workload can be reduced via crowdsourcing,^36^ if the research community is willing and able to provide relevant data, at best in the desired format. To support users in the submission of data, we plan to use the submission assistant of PsychArchives (https://www.psycharchives.org/). To ensure interoperability of the data with PsychOpen CAMA for the implementation on the platform, manual effort for validity checks will still be needed. We will automate repetitive processes as far as possible, for example by using notifications in case of new data entries, and scripts for validity checks. But at least for the monitoring of these processes, additional plausibility checks, and necessary corrections in case of erroneous entries, manual effort cannot fully be replaced.

To strategically acquire new data for PsychOpen CAMA, there are more resources to be used. Research data from primary studies shared in PsychArchives can be used to update corresponding meta‐analyses in CAMA. Alternatively, the results of studies or even complete meta‐analyses preregistered at ZPID (https://prereg-psych.org/), as well as data collected in ZPID's online or offline laboratory will be used to extend the database for PsychOpen CAMA. For meta‐analyses published in one of the journals of PsychOpen (https://www.psychopen.eu/), authors could be asked to share the meta‐analytic data of the meta‐analysis. The long‐term goal of these strategies is to automate these linkages as far as possible to accumulate evidence in PsychOpen CAMA and keep pace with the mass of scientific results produced and published in various domains in psychology.

### 5.2 Analytical and methodological scope

To make data use for further analyses easier, PsychOpen CAMA will be connected to PsychNotebook (freely available here: https://www.psychnotebook.org/), a cloud‐based notebook for statistical analyses in psychology and related disciplines. Advanced users interested in applications that go beyond those directly available on the GUI of PsychOpen CAMA may use the meta‐analytic datasets within the free R environment in PsychNotebook. Furthermore, commented code snippets for advanced meta‐analytic functionalities will be provided to facilitate the analyses in PsychNotebook, and to serve educational purposes. Users can create their own projects within PsychNotebook, where they can collect and save their own ideas, analysis scripts and outputs and share these with others.

There are various approaches in meta‐analyses. For comparing multiple treatments in clinical psychology^37^ or the effects of interventions on behavior,^38^ network meta‐analysis is of particular importance.^39^ For the estimation of overall mean estimates and interactions, the combination of available individual participant data (IPD) with aggregate data (AD) is desirable,^40^ suggesting the use of available raw data from studies included in meta‐analyses whenever available in data archives. In behavioral and social sciences, relationships are often represented in the form of complex models including relations between several variables simultaneously. The results of structural equation models used to depict those relationships can be meta‐analyzed with the help of the MASEM approach.^41^

All these aforementioned approaches require data standards and analysis outputs different from those already available in PsychOpen CAMA. Data templates and the implementation of special analysis functionalities for these kinds of meta‐analyses are therefore needed to broaden the scope of the platform.

## 6 DISCUSSION

With a growing number of publications, the survival time of synthesized evidence is short. Efficient accumulation and synthesis of knowledge becomes the key to making scientific results usable and valid. To keep meta‐analyses up to date, they have to be published in a format allowing the reuse of data and an easy avenue to verify, modify, and update meta‐analyses.

PsychOpen CAMA is presented as a solution to enable dynamic and reusable meta‐analyses. It provides a repository for interoperable meta‐analytic data and a GUI for easy access to the results from the available analyses to the research community and the public. However, challenges regarding automation of workflows, flexibility of analysis options, and the scope of the platform remain. There is great potential to address these challenges by using further resources and tools at ZPID.

## CONFLICT OF INTEREST

The authors declare no conflict of interest.

## Data Availability

All data published in PsychOpen CAMA are available under a CC BY 4.0 license in PsychArchives for download and further use (https://www.psycharchives.org/) and in PsychOpen CAMA (https://cama.psychopen.eu/) for use in the application. The data template for meta‐analyses is also available on the platform. The self‐maintained R package used for the outputs in the application is available and documented in the following Git repository under a GPL‐3.0 License: https://github.com/leibniz-psychology/PsychOpen-CAMA-R-package.
